# Do composition and diversity of bacterial communities and abiotic conditions of spring water reflect characteristics of groundwater ecosystems exposed to different agricultural activities?

**DOI:** 10.1002/mbo3.681

**Published:** 2018-07-13

**Authors:** Karsten Karczewski, Patricia Göbel, Elisabeth I. Meyer

**Affiliations:** ^1^ Institute for Evolution and Biodiversity University of Münster Münster Germany; ^2^ Institute of Geology and Palaeontology University of Münster Münster Germany

**Keywords:** anthropogenic activities, bacterial community composition, bacterial diversity, underground spring catchment area

## Abstract

Modern agricultural practices have undeniably increased global food production. On the other hand, agricultural practices not only lead to a degradation of natural ecosystems but also affect the functioning of ecosystems and the related services they provide. Even though impacts of anthropogenic activities vary across ecosystems, freshwater ecosystems are among those affected to a higher degree. In comparison to surface water ecosystems, groundwater ecosystems are less affected by anthropogenic pollutants, as the overlaying soil retains organic and inorganic substances. However, it has become evident that the excessive use of fertilizers has led to the eutrophication of many aquifers. Bacterial communities, which significantly contribute to the cycling of matter due to their metabolic capacities, are prone to environmental perturbations, and structural variation of bacterial communities may consequently affect the functioning of groundwater ecosystems. Our present paper intends to evaluate the impact of anthropogenic activities on environmental conditions as well as on the structural properties of bacterial communities in groundwater. We repeatedly sampled emerging groundwater at five spring sites belonging to different catchments and determined the concentration of abiotic variables as well as the diversity and composition of bacterial communities on a local scale. We hypothesized that anthropogenic activities influence the concentration of abiotic variables, especially of nitrate, as well as the composition and diversity of bacterial communities in groundwater. Our results show that underground spring catchment areas only slightly differ regarding the concentration of abiotic variables as well as the structure of bacterial communities. Furthermore, abiotic variables, presumably influenced by anthropogenic activities, do not correlate with the diversity and composition of bacterial communities. Although supported only by circumstantial evidence, we suggest that upwelling groundwater from the deeper aquifer affects the diversity and composition of bacterial communities, and we argue that bacterial communities act as useful indicators for environmental changes.

## 1 INTRODUCTION

Global food production has been successfully increased due to the conversion of natural landscapes for agricultural usage in combination with modern agricultural land use practices (Foley et al., 2005). However, agriculture influences the composition and functioning of ecosystems directly and indirectly, resulting in a degradation of ecosystems and their related services, some of which are also relevant for agriculture (Foley et al., 2005; Tilman et al., 2001). Eutrophication, caused by the excessive application of fertilizers, is one of the major threats that alters the composition and functioning of ecosystems (Tilman et al., 2001). Even though the impacts of eutrophication vary in severity between ecosystems, freshwater environments are considered to be more greatly affected than terrestrial ecosystems (Dudgeon et al., 2006).

Groundwater, which comprises 96% of the usable freshwater (Shiklomanov & Rodda, 2004), is one of the world's most extracted renewable resources for agricultural, industrial and domestic purposes (Morris et al., 2003). The exploitation of groundwater has expanded since the middle of the 20th century due to advances in geological knowledge, well drilling, pump technology, and rural electrification (Foster & Chilton, 2003). The extraction of groundwater has certain advantages in comparison to surface water; the drought resilience of groundwater allows extraction during dry seasons or during long interannual droughts, and additionally the spatial extension of groundwater enables local extraction (Döll, 2009; Kundzewicz & Döll, 2009; Morris et al., 2003). Most importantly, groundwater aquifers generally exhibit longer water retention times, fostering natural attenuation (Foster & Chilton, 2003; Haag & Kaupenjohann, 2001; Morris et al., 2003; Scow & Hicks, 2005) which is why groundwater is considered to be less vulnerable to anthropogenic pollutants than surface waters (Foster & Chilton, 2003).

However, the vulnerability of groundwater may vary and is generally influenced by aquifer type, permeability, and chemical characteristics (Morris et al., 2003). With regard to these characteristics, aquifers may act as conduits or retention compartments, both affecting natural attenuation (Haag & Kaupenjohann, 2001). Water quality improvement is a complex process involving manifold factors that together drive the attenuation process. For example, nitrate, a major contaminant in groundwater (Böhlke, 2002), can be substantially removed in retention compartments within the aquifer due to denitrification processes (Böhlke, 2002; Haag & Kaupenjohann, 2001; Rivett, Buss, Morgan, Smith, & Bemment, 2008). High concentrations of nitrate in groundwater by comparison can be attributed to the excessive application of fertilizers in areas of intensive agricultural land use (Böhlke, 2002). Since the capacity of the soil to retain nitrogen is often exceeded (Böhlke, 2002; Haag & Kaupenjohann, 2001), nitrate can reach groundwater aquifers in vast amounts, resulting in a degradation of groundwater quality (Peters & Meybeck, 2000). Apart from alteration of the abiotic environment, eutrophication is likely to influence the taxonomic composition of bacterial communities. These alterations are often induced by interspecific and intraspecific interaction. Since microorganisms constantly compete for resources, an increase in nutritional resources may foster competition among species (Hibbing, Fuqua, Parsek, & Peterson, 2010), resulting in a loss of species that have been outcompeted by others (Kotsyurbenko, Glagolev, Nozhevnikova, & Conrad, 2001; Portal‐Celhay & Blaser, 2012). Due to the removal of one or several fractions from the community, opportunistic community members may increase in abundance and thus shape the community composition (Costello, Stagaman, Dethlefsen, Bohannan, & Relman, 2012; Lawrence et al., 2012). Apart from competition, species composition can change because species fail to adapt to the newly established environmental conditions (Lawrence et al., 2012). Although tested under controlled laboratory conditions, Lawrence et al. (2012) showed that co‐occurring species were able to modify their environment and thus altered the selection pressure on other species.

The alteration of species composition poses a problem insofar as microorganisms play a dominant role in the natural attenuation of contaminants in groundwater ecosystems (Balke & Griebler, 2003), and it was shown that a reduction in microbial diversity can, for example, affect the cycling of nitrogen in soils (Philippot et al., 2009). The ability of microorganisms to metabolize and biodegrade pollutants is based on the variety of metabolic functions (functional diversity) performed by microorganisms (Danielopol, Griebler, Gunatilaka, & Notenboom, 2003; Goldscheider, Hunkeler, & Rossi, 2006), which provides and sustains high groundwater quality. Although it has yet to be resolved to what extent taxonomic diversity can alter functional diversity (Griffiths et al., 2000; Petchey & Gaston, 2002), it is assumed that ecosystem functioning in general is affected by changes in taxonomic diversity (Díaz, Symstad, Chapin, Wardle, & Huenneke, 2003; Graham et al., 2016).

The aim of this paper was to compare the composition and diversity of bacterial communities as well as associated abiotic variables of underground spring catchment areas on a local scale. We hypothesized (1) that the concentration of abiotic variables, especially the concentration of nitrate, which we suggest to indicate anthropogenic activities, differs between sampling sites. We furthermore hypothesized that (2) bacterial diversity estimates differ between sampling sites in relation to differences in abiotic variables, and (3) bacterial community composition (BCC) differs between underground spring catchment areas and is shaped by abiotic variables.

## 2 METHODS

### 2.1 Study area

**Figure 1:**
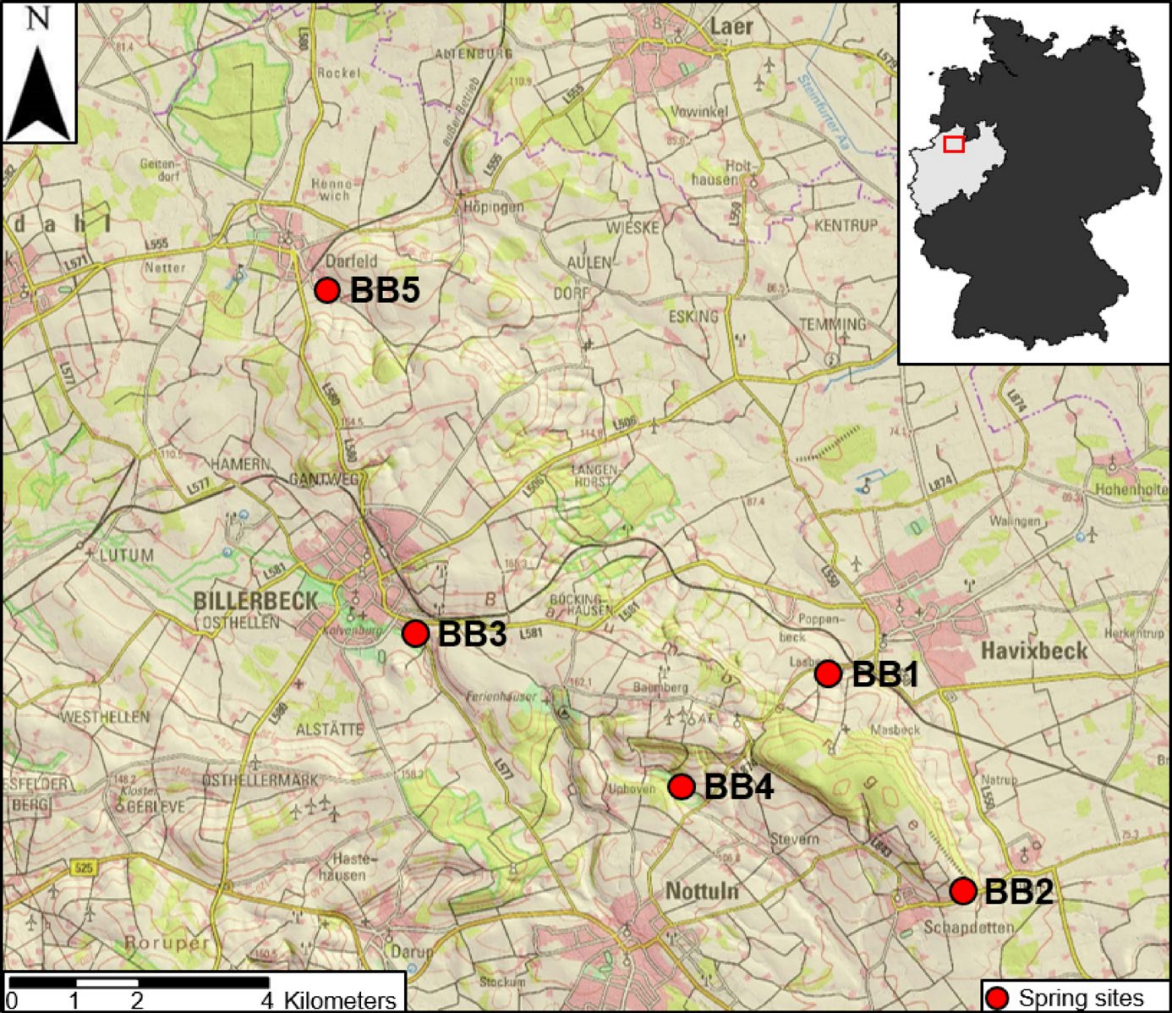
Geographic location of sampling sites. Germany is indicated in black in the inset map, and the state of North Rhine‐Westphalia in pale gray. The region where the sampling was done is shown as a red rectangle. Sampling sites are indicated by the red dots

The study area is a hilly region (max. elevation + 187 m·a·s·l) called “Baumberge” (BB) located west of the city of Münster, situated in North Rhine‐Westphalia, Germany (Figure 1). The geology of the study area is characterized by deposits of the upper Cretaceous and can be divided into two layers. The upper layer (“Baumberge layer”) consists of sand marl and lime marl stones, and due to its crevasse formation and porosity, is permeable to water. The lower layer (“Coesfeld layer”) consists of clay and lime marl stones, and is less permeable to water. In the subsurface, the “Coesfeld layer” exhibits a bowl‐like structure. The study area is the highest elevation in the otherwise flat landscape of the Westphalian Lowland and represents a precipitation barrier. Rain water seeps through the fissured “Baumberge layer” and accumulates on top of the lower “Coesfeld layer”. The rain water circulates in the porous aquifer and emerges in the form of springs at the edge of the “Coesfeld layer” (Göbel, 2010). The study area can furthermore be considered a hydrographical knot, as springs in the area are tributaries belonging to the adjacent catchment areas of five distinct streams: “Münstersche Aa”, “Stever”, “Steinfurter Aa”, “Berkel”, and “Vechte” (Düspohl & Messer, 2010). We sampled five springs of four distinct catchment areas in October 2014, December 2014, January 2015, and March 2015 (Table 1). The sampling sites exhibit similar geological characteristics, and previous analyses of water samples revealed only slight differences in the concentration of main chemical components. Analyses furthermore showed that the springs are anthropogenically influenced, indicated by varying and elevated nitrate concentrations, suggesting the influence of agricultural land use (Hafouzov, 2010).

**Table 1:** Elevation, spring types, and geographical coordinates of the five sampled springs in the Baumberge region

Spring	Catchment	Elevation	Spring type	Latitude	Longitude
BB1	Münstersche Aa	109 m·a·s·l	Helocrene	51.96655	7.39767
BB2	Stever	97 m·a·s·l	Rheocrene	51.93665	7.42922
BB3	Berkel	115 m·a·s·l	Limnocrene	51.97092	7.39767
BB4	Stever	110 m·a·s·l	Rheocrene	51.95069	7.36552
BB5	Vechte	102 m·a·s·l	Rheocrene	52.01893	7.28250

### 2.2 Spring water sampling

In total, spring water samples were collected at five locations (BB1‐BB5) at four different points in time. The sample names are based on the location as well as on the time of collection (e.g. BB1.1: Site BB1 at time point 1). Two water samples were collected at each sampling point. The first water sample (1 L) was used for the analyses of microbial community structure, and the second water sample (200 ml) was used for the analyses of abiotic parameters. Simultaneously to water sample collection, in situ parameters of spring water (temperature (T), electric conductivity (EC), pH‐value, and dissolved oxygen concentration (DO)) were measured in a beaker using field measurement equipment (Cond 3310, pH 3310, Oxi 3310, WTW, Germany). Samples were extracted using a hand pump and collected in autoclaved glass bottles. All devices were rinsed three times with 400 ml of spring water prior to sample collection. All water samples were kept on ice and in the dark until further processing in the laboratory on the same day.

### 2.3 Analysis of abiotic conditions

The concentration of abiotic variables was measured directly after retrieval of samples to the laboratory. The concentration of potassium (K^+^), sodium (Na^+^), strontium (Sr^2+^), magnesium (Mg^2+^), calcium (Ca^2+^), and aluminum (Al^3+^) in water samples was measured by means of inductively coupled plasma optical emission spectrometry (ICP‐OES) using a SpectroFlame‐EOP (SPECTRO Analytical Instruments GmbH, Germany). Samples were acidified with nitric acid prior to determination. For the measurement of fluoride (F^−^), nitrate (NO_3_^−^), chloride (Cl^−^), sulfate (SO_4_^2−^), and phosphate (PO_4_^3−^), water samples were filtered through a 0.45 μm pore size membrane filter and subsequently analyzed by means of ion chromatography using a 761 compact IC (Methrom AG, Switzerland). In order to determine the concentration of silicate anions (SiO_3_^2−^), we measured the Si^4+^ cations using ICP‐OES, and subsequently calculated the concentration of SiO_3_^2−^ anions using a conversion factor of 2.71. The concentration of hydrogen carbonate (HCO_3_^−^) was determined by means of titration using hydrogen chloride (HCl). Dissolved organic carbon (DOC) was measured using a TOC‐LCPH/LCPN‐Analyzer (Shimadzu Corporation, Japan), using the procedure via nonpurgeable organic carbon (NPOC). All measurements were conducted following standardized norms. Since the concentrations of fluoride, phosphate, and aluminum were below the limit of detection for the majority of samples, these parameters were excluded for further analyses as they could not be analyzed statistically.

### 2.4 DNA extraction

DNA was extracted as detailed in Karczewski, Riss, and Meyer (2017). In brief, to concentrate bacterial cells, water samples were filtered individually through a 0.2 μm pore size sterile membrane filter (Whatman, UK) using a vacuum pump (Vacuubrand GMBH & Co., Germany). The PowerWater^®^ DNA isolation kit (Mo Bio Laboratories, Inc., USA) was afterward used to isolate total DNA from the filter, following the manufacturer's protocol. An amount of 30 μl of elution buffer (10 mM Tris‐HCl EDTA) was used to elute the isolated DNA. Samples were stored at −20°C until further processing.

### 2.5 DNA amplification

Isolated DNA of each sample served as a template for polymerase chain reaction (PCR), carried out using a Mastercycler^®^ nexus PCR system (Eppendorf, Germany). The universal bacterial primers 799F (5′‐AAC MGG ATT AGA TAC CCK G‐3′) and 1114R (5′‐GGG TTG CGC TCG TTG‐3′) (Hanshew, Mason, Raffa, & Currie, 2013) were used to ensure the amplification of 300 base pair bacterial 16S rRNA gene sequences. A mastermix containing 5 μl PCR‐buffer (5x Green GoTaq Flexi Buffer, Promega, USA), 2.5 mM MgCl_2_ (Promega, USA), 0.48 mM dNTPs (0.12 mM each), 0.1 pmol of each primer (MetaBion, Germany), and 0.025 U GoTaq polymerase (Promega, USA) was prepared and added up with sterile double distilled water to give a final volume 24 μl for each PCR reaction. Finally, 1 μl of isolated DNA (10 ng) was added to each reaction. For the PCR, the following conditions were used: initial denaturation for 2 min at 94°C followed by 30 cycles of denaturation at 94°C for 20 s, annealing at 50°C for 20 s, extension at 72°C for 45 s, and a final extension of 10 min at 72°C. Each sample was amplified in triplicate. Subsequently, the performance of the amplification was verified by gel electrophoresis using 7 μl of PCR product on an agarose TBE gel (1% w/v) using Roti^®^‐GelStain (Carl Roth GmbH & Co. KG, Germany). Positive PCR products of the same sample were pooled afterward and purified using the GeneJet PCR Purification Kit (Thermo Fisher Scientific Inc., USA) according to the manufacturer's protocol. Samples were eluted in 30 μl of Tris‐EDTA buffer (pH 8) and quantified using the Qubit^®^2.0 Fluorometer (Thermo Fisher Scientific Inc., USA).

### 2.6 High‐throughput sequencing (HTS)

High‐throughput sequencing (HTS) of amplified 16S rRNA gene sequences was carried out using the Ion PGM^™^ Template OT2 200 Kit (Thermo Fisher Scientific Inc., USA) according to the manufacturer's protocol. A Bioanalyzer 2100 (Agilent Technologies, USA) was used to ensure that amplification of isolated DNA yielded amplicons of 300 base pairs as well as to measure the concentration of the amplicons. The Ion Xpress^™^ Template 200 Kit (Thermo Fisher Scientific Inc., USA) was used accordingly to the manufacturer's protocol to attach Ion Sphere Particles (ISP) to the samples. Prior to the attachment, each sample was brought to a final concentration of 26 pM. Barcoded libraries were pipetted on an Ion 316^™^ v2 Chip (Thermo Fisher Scientific Inc., USA) and subsequently sequenced using an Ion Torrent TM Personal Genome Machine TM (PGM) (Thermo Fisher Scientific Inc., USA). Sequencing data were exported as FastQ files after removal of low quality and polyclonal sequences within the PGM software, and afterward analyzed using the software MOTHUR (Schloss et al., 2009), following the method described by Schloss, Gevers, and Westcott (2011). The SILVA rRNA database v. 123 (Quast et al., 2013) was used to align and classify bacterial 16S rRNA gene sequences taxonomically by genera. Sequences were grouped into operational taxonomic units (OTU) on a basis of 97% similarity, resulting in a data matrix containing OTU abundance‐by‐sample.

### 2.7 Statistical analyses

All statistical analyses were conducted using R v.3.2.3 (R Core Team, 2015). In total, data from 20 samples were analyzed using the packages “phyloseq” v. 1.14.0 (McMurdie & Holmes, 2013), “BiodiversityR” v. 2.7‐1 (Kindt & Coe, 2005), “vegan” v. 2.3‐5 (Oksanen et al., 2007), “ape” v. 3.4 (Paradis, Claude, & Strimmer, 2004), “car” v. 2.1‐4 (Fox & Weisberg, 2011), and “multcomp” v.1.4‐6 (Hothorn, Bretz, & Westfall, 2008). In order to evaluate differences in environmental conditions, individual samples were first assigned to groups according to their sampling site. Subsequently, we generated generalized linear models (GLM) for each abiotic variable individually, and evaluated goodness of fit for the allocation of samples to sampling site as a criterion. We assessed bacterial diversity of each sample individually by calculating the number of OTUs present per sample (S), Shannon (H′) and Simpson index (α), and Pielou's evenness (J′), for the OTU abundance‐by‐sample matrix. Differences in diversity estimates between sampling sites was tested by combining individual samples according to their sampling site. GLMs were generated afterward for each diversity estimate individually and evaluated goodness of fit for the allocation of samples to sampling site as a criterion. BCC of individual samples was determined by initially calculating Bray–Curtis dissimilarities (Bray & Curtis, 1957) for the OTU abundance‐by‐sample matrix. The results were subsequently ordinated using principal coordinates analysis (PcoA) (Gower, 1966), and goodness of fit for the assignment of samples to the corresponding sampling site was tested using permutational multivariate analysis of variance (PERMANOVA) (Anderson, 2001). Interactions between BCC and environmental conditions were tested by correlating measured environmental variables with calculated Bray–Curtis dissimilarities.

## 3 RESULTS

### 3.1 Abiotic conditions

The results of abiotic conditions reveal that five of the fifteen abiotic variables differ significantly in concentration between the five sampling sites notably for variables strontium, magnesium, nitrate, and sulfate (Table 2).

**Table 2:** The effect of sampling site on the concentration of measured abiotic parameters

Abiotic parameter	Sum of squares	*F*‐ratio	*p*‐value
T	0.72	0.15	0.961
pH	6.91	2.14	0.125
EC	5.57	1.55	0.237
DO	3.19	0.76	0.568
DOC	4.70	1.23	0.339
K^+^	8.07	2.77	0.066
Na^+^	2.55	0.58	0.680
Sr^2+^	17.07	33.22	<**0.001**
Mg^2+^	15.11	14.56	<**0.001**
Ca^2+^	5.83	1.66	0.211
HCO_3_^−^	8.58	3.09	**0.049**
SiO_3_^2−^	0.39	0.08	0.988
NO_3_^−^	14.95	13.84	<**0.001**
Cl^−^	4.31	1.10	0.393
SO_4_^2−^	12.90	7.93	**0.001**

*Notes*. Degrees of freedom for all tests is 4. Numbers in bold indicate a significant effect of sample site.

**Figure 2:**
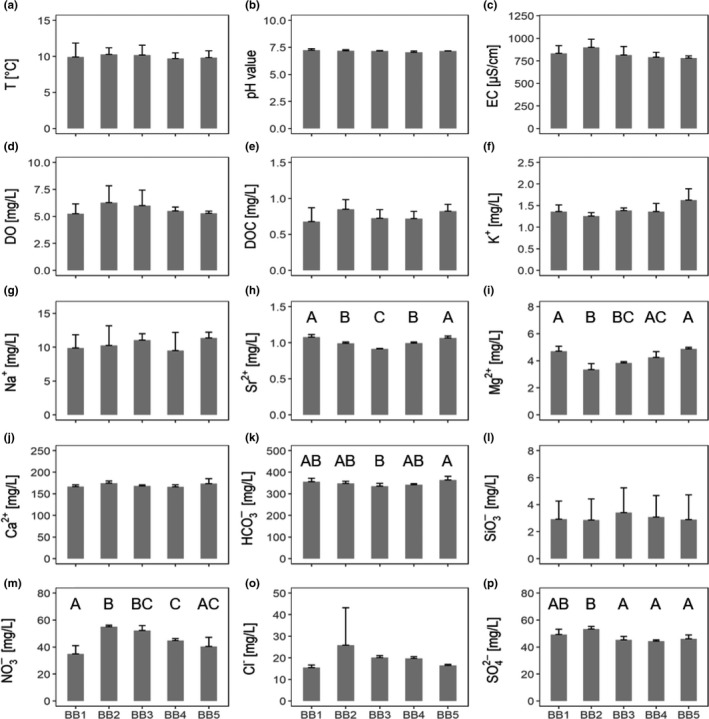
(A–P) Environmental variables measured at each of the five sampling sites indicated on the *x*‐axis. Bars show means plus 1 standard deviation with *n* = 4. Where letters above the mean values for a variable are not the same, it indicates that the means are significantly different from each other. There was no global effect of sampling site on panels containing no letters. For location of the sampling sites, see Table 1

Differences in variables are especially evident between sampling sites BB3 and BB5 (Figure 2H, I, K, and M), and springs BB2 and BB5 (Figure 2H, I, M, and P). Despite significant differences of abiotic variables across sampling sites, underground catchment areas do not differ consistently regarding concentrations of abiotic variables. For example, sampling sites BB2 and BB3 share a similar concentration regarding nitrate (Figure 2M), but differ significantly in variables strontium, and sulfate (Figure 2H and P). No significant differences exist between sampling sites BB1 and BB5 (Figure 2H, I, M, and P).

Additionally, we found a significant positive correlation between variables strontium and magnesium (*p *=* *0.006; Rho: 0.65; Spearman's rank correlation), a negative correlation between strontium and nitrate (*p *=* *0.002; Rho: −0.70; Spearman's rank correlation), and a negative correlation between magnesium and nitrate (*p* < 0.001; Rho: −0.80; Spearman's rank correlation) (Table 3).

**Table 3:** Correlation of abiotic variables showing differences across sampling sites

Variable	Mg^2+^	HCO_3_^−^	NO_3_^−^	SO_4_^2−^
Sr^2+^	**0.65** *	0.48	−**0.72** **	0.17
Mg^2+^		0.23	−**0.81** ***	−0.17
HCO_3_^−^			−0.28	−0.07
NO_3_^−^				0.09

*Notes*. Numbers in bold indicate a significant correlation (*p < 0.05, **p < 0.005, ***p < 0.001).

### 3.2 Bacterial diversity

For the comparison of bacterial diversity, a total of 3,375,180 raw sequences were obtained from a total of 20 samples, with a mean sequence number of 168,759 (±79,083 *SD*) per sample. After conducting quality filtering, trimming, and removal of chimeric sequences, it resulted in a total of 322,847 sequences with a mean of 16,142 (±8,439 *SD*) per sample. Differences in sequencing depth were accounted for by normalizing sequences to a total of 3,684, which was the minimum number of sequences found in sample BB3.1. The final dataset comprised of 1,906 OTUs and a total of 73,680 sequences.

**Figure 3:**
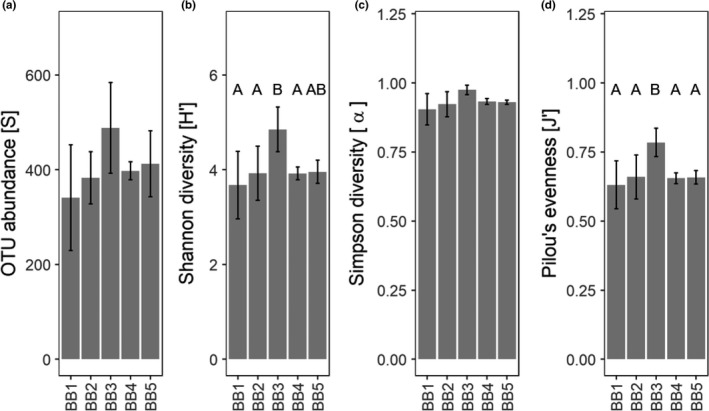
(A–D) Diversity estimates per sampling site calculated from operational taxonomic units (OTUs) recovered by HTS. Sampling sites are indicated on the *x*‐axis and bars show means from four sampling dates plus minus 1 standard deviation. Where letters above the mean values for a parameter are not the same, it indicates that the means are significantly different from each other. There was no global effect of sampling site on panels containing no letters

The comparison of calculated diversity estimates (Figure 3) shows that sampling sites differ significantly in the expression of Shannon diversity and Pielou's evenness (Table 4; Figure 3B and D). However, sampling sites do not differ significantly regarding OTU abundance and Simpson diversity index (Table 4). The results show a tendency for sampling site BB3 to harbor the highest Shannon diversity and differ significantly from the other springs with the exception of BB2 (Figure 3B). Furthermore, sampling site BB3 has the highest value for Pielou's evenness, and differs significantly from all other sampling sites (Figure 3D).

**Table 4:** The effect of sampling site on calculated bacterial diversity estimates

Diversity estimate	Sum of squares	*F*‐ratio	*p*‐value
OTU abundance	6.48	1.94	0.156
Shannon diversity	9.32	3.61	**0.030**
Simpson diversity	7.33	2.36	0.102
Pielou's evenness	10.04	4.20	**0.018**

Notes. Numbers in bold indicate a significant effect of sampling site.

### 3.3 Relative taxonomic composition

**Figure 4:**
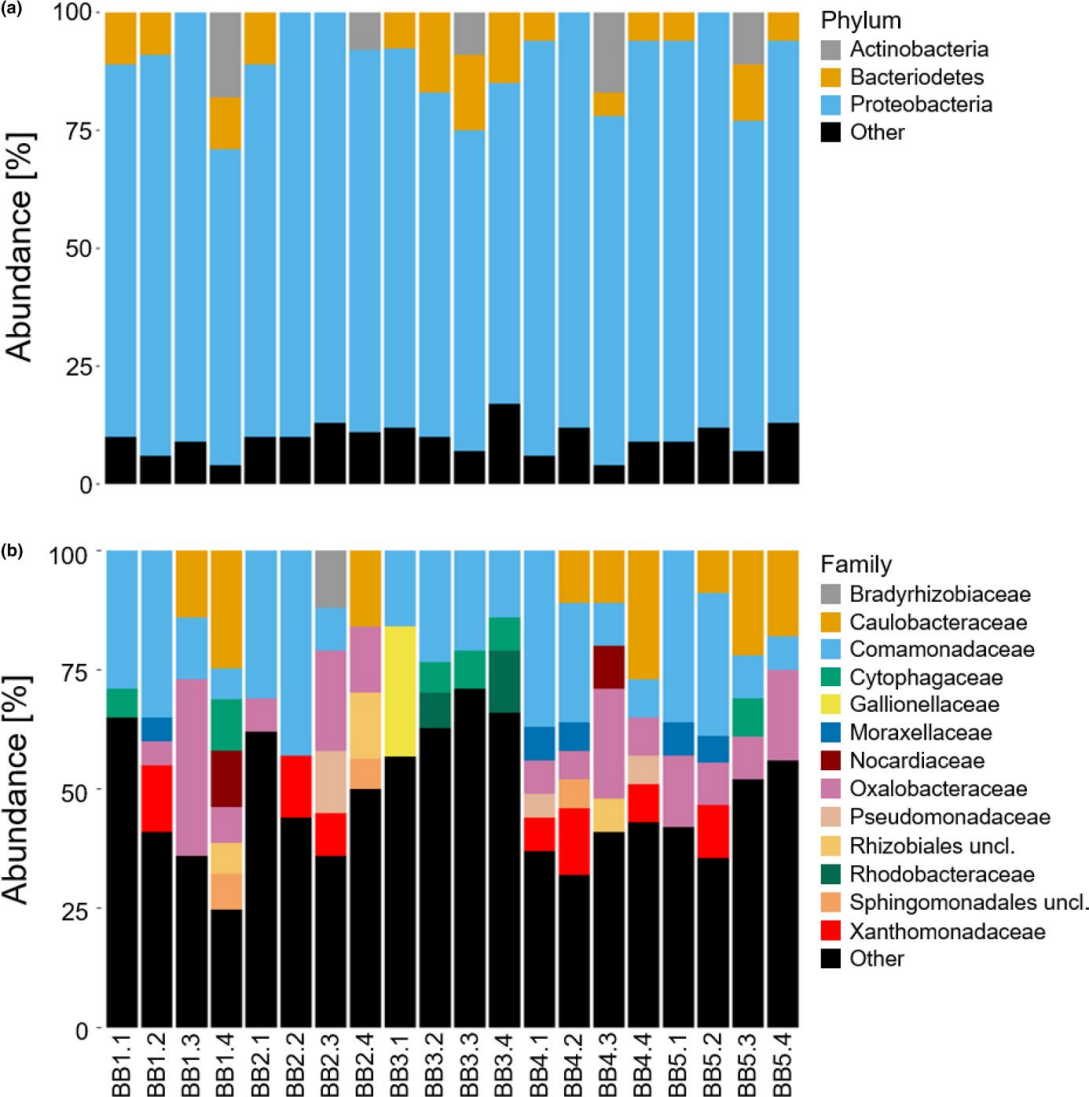
(A and B) Relative bacterial abundance of samples at different taxonomic levels. The legends to the right of the figures show the dominant taxa, i.e. those with a relative individual abundance >5%, and rare taxa, i.e. those with a relative individual abundance <5% (“Other”). Sampling site information is indicated on the *x*‐axis. For location of the sites, see Table 1. The first number indicates the sampling site, the second number refers to time of sampling

The majority of sequences obtained from each sampling site is assigned to the Proteobacteria phylum (80%) (Figure 4A). The proportion of rare phyla, which is phyla with an individual relative abundance of less than 5% (represented by “Other”, Figure 4A and B) compose on average 9.5% of the overall abundance. While sampling sites display similar patterns in relative taxonomic composition at the phylum level, a more specific pattern can be seen at the family level (Figure 4B). On the family level, Comamonadaceae is the most abundant bacterial family across sampling sites (19%) (Figure 4B). Rare families compose a considerable average proportion of 47% of the overall abundance and exceeded 70% of the proportion of sample BB3.3 (Figure 4B). Sampling site BB3 in particular showed a distinct taxonomic composition pattern, whereby the more common families, i.e. Oxalobacteraceae, Caulobacteraceae, and Xanthomonadaceae were not observed in any samples. This pattern extended to some of the more scarce families, Nocardiaceae, Moraxellaceae, Rhizobiales_unclassified, Sphingomonadales_unclassified, Bradyrhizobiaceae, and Pseudomonadaceae, which were also not present in BB3. The site furthermore tends to exhibit on average the highest proportion of rare families (61%), but only differs in this regard from sampling site BB4 (*p *=* *0.03; Wilcoxon rank sum test).

### 3.4 Bacterial community composition

**Figure 5:**
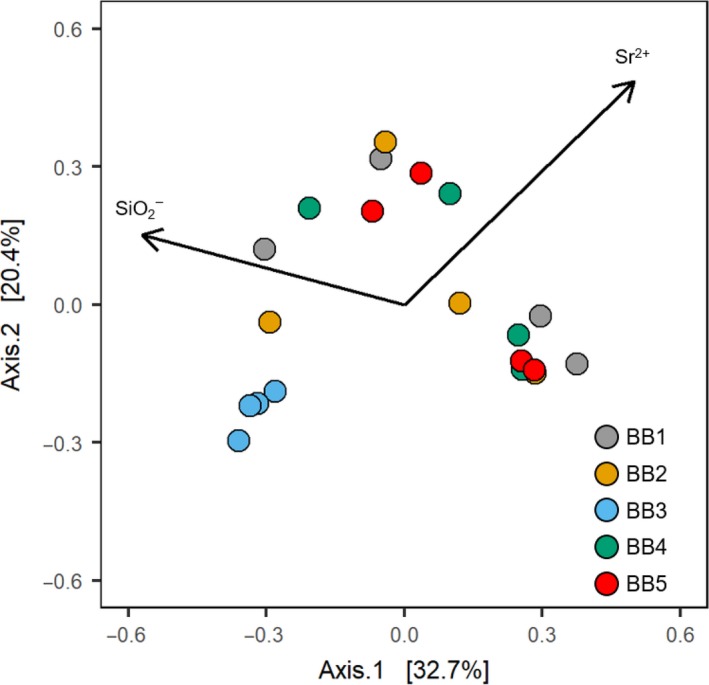
Principal coordinates analysis (PCoA) of recovered OTUs. Depicted are site scores of each sample, as well fitted environmental variables with significant correlations (*p* < 0.05). Angle and length of vectors is proportional to the direction and strength of the abiotic variable, and the direction of the arrows

We identified a significant effect of sampling site on the variation of BCC (*p *=* *0.031; PERMANOVA), although site only accounts for 31% of the variation across springs. Differences in bacterial taxonomic composition between spring BB3 and the other springs are also revealed by analysis of principal coordinates (Figure 5). While samples of sites BB1, BB2, BB4, and BB5 do not display site‐specific BCCs, samples from site BB3 exhibit a similar community composition across sampling times (Figure 5). The correlation of abiotic variables against the ordination plot revealed that the discriminating factors of BCC for the sampling sites can be mainly explained by strontium and silicate (Figure 5; Table 5).

**Table 5:** Correlation of abiotic variables to Bray–Curtis dissimilarities of OTU composition of sampling samples (see Table 1)

Abiotic variable	*R* ^2^	*p*‐value
T	0.142	0.266
pH	0.052	0.642
EC	0.306	0.054
DO	0.005	0.962
DOC	0.174	0.200
K^+^	0.059	0.564
Na^+^	0.023	0.819
Sr^2+^	0.485	**0.005**
Mg^2+^	0.271	0.079
Ca^2+^	0.013	0.900
HCO_3_^−^	0.129	0.283
SiO_3_^−^	0.347	**0.027**
NO_3_^−^	0.267	0.074
Cl^−^	0.063	0.757
SO_4_^2−^	0.116	0.342

Notes. Numbers in bold indicate a significant correlation.

## 4 DISCUSSION

In this study, we collected water from five distinct sampling sites with similar geological characteristics in underground spring catchment area. We tested whether measured abiotic variables as well as the diversity and composition of microbial communities differ between sampling sites, and whether microbial communities reflect the differences in abiotic variables.

The concentrations of abiotic variables were generally within the range of groundwater of similar geological composition (Bakalowicz, 1994). Additionally, the concentrations of the majority of measured abiotic variables did not differ across sampling sites, and correspond to findings of previous studies in the region (Hafouzov, 2010). Since spring water is fed by groundwater from underground spring catchment areas, and since the main chemical characteristics of groundwater are related to aquifer geochemistry (Bakalowicz, 1994; Griebler & Mösslacher, 2003), our results indicate the influence of similar hydrological conditions. However, five abiotic variables differed across sampling sites predominantly for the variables strontium, magnesium, nitrate, and sulfate (Table 2). Differences in the concentration of and variation in nitrate across sampling sites were previously identified, suggesting the influence of agricultural land use (Hafouzov, 2010). Elevated nitrate concentrations are known to result from agricultural land use practices (Bakalowicz, 1994), and differences might result either from varying intensities of agricultural land use practices, varying sizes of arable land, varying sizes of underground spring catchment areas, or differences in soil properties affecting leaching of nitrate into the groundwater (Lockhart, King, & Harter, 2013; McLay, Dragten, Sparling, & Selvarajah, 2001; Wick, Heumesser, & Schmid, 2012). Strontium might indicate upwelling groundwater (Hafouzov, 2010) and shows the highest concentrations at sampling sites BB1 and BB5, differing significantly from the other three sampling sites. The results thus partly support our hypothesis (1) that underground spring catchment area characteristics would only differ in those abiotic variables that are influenced by anthropogenic activities.

Regarding our hypothesis (2) that bacterial diversity differs between sampling sites in relation to differences in abiotic variables, we found that differences in bacterial community diversity were only evident for spring BB3, and only for the parameters Shannon diversity and Pielou's evenness. Site BB3 displayed the highest bacterial diversity as well as the most even composition of bacterial OTUs (Figure 3B and D). However, it is important to highlight that the bacterial diversity of each sampling site was obtained by combining four individual samples from different time points, and the results may thereby inadequately represent the bacterial diversity of the sampling sites. Despite this potential drawback, similarly to the diversity estimates, relative BCC also showed only few differences at the phylum level. All samples are dominated by the phylum Proteobacteria, a result that corresponds to observations reported from other studies (Braun, Schröder, Knecht, & Szewzyk, 2016; Wright et al., 2017). Due to the fact that geochemical characteristics are the main drivers of BCC (Constancias et al., 2015), and since geochemical characteristics differ only slightly across sampling sites, we suggest that this results in a similar taxonomic community composition at the phylum level. This was not unexpected and confirms observations reported by Staley and Sadowsky (2016) that variation in community composition tends to be smaller within the same region. However, differences in taxonomic community composition between sampling sites were more apparent at the family level. In conjunction with the results of bacterial diversity, it is most likely that significant differences in diversity estimates across sampling sites are driven by the proportion of rare families, notably for sampling site BB3, where rare families compose approximately 61% of the overall abundance (Figure 4B). The influence of rare families on bacterial diversity is furthermore supported by the fact that diversity differs significantly across sampling sites for Shannon diversity, but not for Simpson's diversity. This finding likely results from the fact that Shannon diversity is equally sensitive to rare and abundant species, whereas Simpson's diversity index is more sensitive to abundant species (Morris et al., 2014; Vuono et al., 2015). According to the intermediate disturbance hypothesis (Connell, 1978), disturbance prevents competitive species from dominating a given habitat while enabling space for colonization of less competitive species (Gibbons et al., 2016), which is why an increase in the number of rare species is indicative of disturbance (Piper, Siciliano, Winsley, & Lamb, 2015). Furthermore, circumstantial support for disturbance is the fact that the disturbance‐tolerant phylum, Bacteriodetes (Kim, Heo, Kang, & Adams, 2013), compose approximately 14% of bacterial communities sampled at spring BB3 (Figure 4A), although differences were found to be significant only in comparison to spring BB4 (*p *=* *0.03; Wilcoxon rank sum test).

Differences between sampling sites regarding bacterial diversity and relative taxonomic abundance are furthermore confirmed by analysis of principal coordinates of bacterial OTU composition at the family level. We found a significant effect of sampling site on OTU composition (*R* ^2^ = 0.311; *p *=* *0.031; PERMANOVA), a result that was previously reported by others (Kaiser et al., 2016; Ma, Ibekwe, Yang, & Crowley, 2016; Tardy et al., 2015). However, the effect of sampling site on OTU composition was low (31%) in comparison to findings reported by Ma et al. (2016) (50.9%), and is only apparent for samples collected at sampling site BB3 (Figure 5). Hence, variation in community composition must be partly accounted for by variables other than sampling site.

The correlation of BCC with measured variables revealed that strontium and silicate are discriminating drivers of community composition across underground spring catchment areas (Table 5; Figure 5). The results therefore support our hypothesis (3) that BCC differs between sampling sites and is shaped by abiotic variables. However, the results were unexpected, as only strontium was identified to differ between underground spring catchment areas, compared to silicate. Furthermore, formerly reported differences in phosphate concentrations (Hafouzov, 2010) cannot be supported by our data. Interestingly, nitrate, which we hypothesized would influence bacterial communities, is negatively correlated with strontium but had no significant effect on either the composition or diversity of bacterial communities, which is in contrast to results reported from other studies (Ben Maamar et al., 2015; Carrino‐Kyker, Smemo, & Burke, 2012; Ibekwe, Ma, & Murinda, 2016; Turlapati et al., 2013; Zhang et al., 2016). Nitrate might thus either not be a discriminating factor for the variation in BCC, or the effect of strontium overrides the effect of nitrate.

A possible explanation for the correlation between strontium and BCC may derive from the fact that the deeper groundwater in the study area exhibits high concentrations of strontium (Hafouzov, 2010). It was shown by previously conducted studies that variations in strontium concentration in spring water were used to indicate the influence of deep groundwater (Bakari et al., 2013; Barbieri, Nigro, & Petitta, 2017; Hofmann & Cartwright, 2013; Liotta, D'Alessandro, Arienzo, & Longo, 2017; Lyons, Tyler, Gaudette, & Long, 1995; Moya, Raiber, Taulis, & Cox, 2016; Shand, Darbyshire, Love, & Edmunds, 2009), and an increase in strontium at the studied sampling sites might therefore indicate upwelling groundwater that mixes with the seeping rainwater. Additionally, bacterial species inhabiting the deeper groundwater mix with the bacteria in the upper layer, forming a newly composed community, a process that has previously been described by Pedersen (2013). Strontium thus acts as a surrogate of upwelling groundwater. Against this background, we suggest that sampling site BB3, which shows a significantly lower concentration of strontium than all other sampling sites, is less influenced by upwelling groundwater, and as a result is less influenced by bacterial communities inhabiting the deeper groundwater. This hypothesis, on the other hand, contrasts with the fact that sampling site BB3 has a similar elevation as the other sites, making it less likely that differences can be explained by elevation alone. Furthermore, altitude and strontium are not correlated (Pearson's product‐moment correlation, *p *>* *0.05), emphasizing the consideration of underground flow paths of the catchment area of sampling site BB3. However, this suggestion cannot be concluded as underground flow paths are to date not yet fully investigated (Schirmer, 2010).

Altogether our results show that the majority of abiotic variables do not differ between sampling sites, indicating similar geological conditions. However, we suggested that the differences observed in a few variables specifically shape bacterial community diversity and composition. As noted above, four temporally spaced samples were combined per sampling site, which may inadequately represent the total bacterial diversity; however, we found that bacterial community diversity was significantly different between sampling sites, and that differences were most apparent only for sampling site BB3. In correspondence with the results obtained from relative taxonomic abundance, differences between sampling sites probably arise due to an increase in the number of rare species. According to Piper et al. (2015), an increase in the number of rare species is an indication of the suppression of dominant species due to disturbance, and hence results in a more even community. Despite differences in nitrate between sampling sites, we did not find a significant effect of nitrate on BCC, which we assumed to be the most indicative variable for disturbance, as it represents the influence of anthropogenic activity. Due to the fact that the number of rare species is especially increased in samples that exhibit low strontium concentrations, and since we provided correlative support for the influence of strontium on the composition of bacterial communities, the results suggest that strontium may be a discriminant driver of community composition. But since strontium has not yet been mentioned to be an important variable for bacterial species, we hypothesize that strontium could also be considered a surrogate for other environmental variables not analyzed in the present paper. Notably, strontium and nitrate are negatively correlated, and it is possible, that the effects of strontium override the effects of nitrate regarding influence on BCC. However, the presented results indicate that the analysis of abiotic environmental variables in combination with the analysis of BCC has the potential to be used to assess environmental conditions as well as to reflect and trace influences that shape the environment. The data we present provide a limited snapshot of the Baumberge springwater bacterial communities; future studies with repeated samples within sampling time, will be necessary to uncover the full extent to which the composition and diversity of these communities vary, both temporally and spatially.

## CONFLICT OF INTEREST

The authors declare no conflict of interest.

## References

[mbo3681-bib-0001] Anderson, M. J. (2001). A new method for non‐parametric multivariate analysis of variance. Austral Ecology, 26, 32–46.

[mbo3681-bib-0002] Bakalowicz, M. (1994). Water geochemistry: Water quality and dynamics In Gibert J., Danielopol D., & Stanford J. A. (Eds.), Groundwater ecology (pp. 97–127). San Diego: Academic Press.

[mbo3681-bib-0003] Bakari, S. S. , Aagaard, P. , Vogt, R. D. , Ruden, F. , Johansen, I. , & Vuai, S. A. (2013). Strontium isotopes as tracers for quantifying mixing of groundwater in the alluvial plain of a coastal watershed, south‐eastern Tanzania. Journal of Geochemical Exploration, 130, 1–14. 10.1016/j.gexplo.2013.02.008

[mbo3681-bib-0004] Balke, K. , & Griebler, C. (2003). Grundwasser Nutzung und Grundwasserschutz In Griebler C., & Mösslacher F. (Eds.), Grundwasser‐Ökologie (pp. 311–365). Vienna: Facultas UTB.

[mbo3681-bib-0005] Barbieri, M. , Nigro, A. , & Petitta, M. (2017). Groundwater mixing in the discharge area of San Vittorino Plain (Central Italy): Geochemical characterization and implication for drinking uses. Environmental Earth Sciences, 76, 393 10.1007/s12665-017-6719-1

[mbo3681-bib-0006] Ben Maamar, S. , Aquilina, L. , Quaiser, A. , Pauwels, H. , Michon‐Coudouel, S. , Vergnaud‐Ayraud, V. , … Dufresne, A. (2015). Groundwater isolation governs chemistry and microbial community structure along hydrologic flowpaths. Frontiers in Microbiology, 6, 1457. 26733990 10.3389/fmicb.2015.01457 PMC4686674

[mbo3681-bib-0007] Böhlke, J. K. (2002). Groundwater recharge and agricultural contamination. Hydrogeology Journal, 10, 153–179. 10.1007/s10040-001-0183-3

[mbo3681-bib-0008] Braun, B. , Schröder, J. , Knecht, H. , & Szewzyk, U. (2016). Unraveling the microbial community of a cold groundwater catchment system. Water Research, 107, 113–126. 10.1016/j.watres.2016.10.040 27837729

[mbo3681-bib-0009] Bray, J. R. , & Curtis, J. T. (1957). An ordination of the upland forest communities of Southern Wisconsin. Ecological Monographs, 27, 326–349.

[mbo3681-bib-0010] Carrino‐Kyker, S. R. , Smemo, K. A. , & Burke, D. J. (2012). The effects of pH change and NO^‐^ _3_ pulse on microbial community structure and function: A vernal pool microcosm study. FEMS Microbiology Ecology, 81, 660–672. 10.1111/j.1574-6941.2012.01397.x 22530997

[mbo3681-bib-0011] Connell, J. H. (1978). Diversity in tropical rain forests and coral reefs ‐ high diversity of trees and corals is maintained only in a non‐equilibrium state. Science, 199, 1302–1310. 10.1126/science.199.4335.1302 17840770

[mbo3681-bib-0012] Constancias, F. , Terrat, S. , Saby, N. P. A. , Horrigue, W. , Villerd, J. , Guillemin, J. P. , … Prévost‐Boure, N. C. (2015). Mapping and determinism of soil microbial community distribution across an agricultural landscape. MicrobiologyOpen, 4, 505–517. 10.1002/mbo3.255 25833770 PMC4475391

[mbo3681-bib-0013] Costello, E. K. , Stagaman, K. , Dethlefsen, L. , Bohannan, B. J. M. , & Relman, D. A. (2012). The application of ecological theory toward an understanding of the human microbiome. Science, 336, 1255–1262. 10.1126/science.1224203 22674335 PMC4208626

[mbo3681-bib-0014] Danielopol, D. L. , Griebler, C. , Gunatilaka, A. , & Notenboom, J. (2003). Present state and future prospects for groundwater ecosystems. Environmental Conservation, 30, 104–130. 10.1017/S0376892903000109

[mbo3681-bib-0015] Díaz, S. , Symstad, A. J. , Chapin, F. S. , Wardle, D. A. , & Huenneke, L. F. (2003). Functional diversity revealed by removal experiments. Trends in Ecology & Evolution, 18, 140–146. 10.1016/S0169-5347(03)00007-7

[mbo3681-bib-0016] Döll, P. (2009). Vulnerability to the impact of climate change on renewable groundwater resources: A global‐scale assessment. Environmental Research Letters, 4(3), 035006 10.1088/1748-9326/4/3/035006

[mbo3681-bib-0017] Dudgeon, D. , Arthington, A. H. , Gessner, M. O. , Kawabata, Z. I. , Knowler, D. J. , Lévêque, C. , … Sullivan, C. A. (2006). Freshwater biodiversity: Importance, threats, status and conservation challenges. Biological Reviews, 81, 163–182. 10.1017/S1464793105006950 16336747

[mbo3681-bib-0018] Düspohl, M. , & Messer, J . (2010). Wasserhaushaltsbilanzierung und grundwasserbürtiger Abfluss in den Baumbergen (Kreis Coesfeld, Nordrhein‐Westfalen) In Göbel P. (Ed.), Abhandlungen aus dem Westfälischen Museum für Naturkunde, 72. Jahrgang, Heft 3/4 (pp. 17–26). Bönen: Druck Verlag Kettler.

[mbo3681-bib-0019] Foley, J. A. , Defries, R. , Asner, G. P. , Barford, C. , Bonan, G. , Carpenter, S. R. , … Snyder, P. K. (2005). Global consequences of land use. Science, 309, 570–574. 10.1126/science.1111772 16040698

[mbo3681-bib-0020] Foster, S. S. D. , & Chilton, P. J. (2003). Groundwater: The processes and global significance of aquifer degradation. Philosophical Transactions of the Royal Society of London Series B‐Biological Sciences, 358, 1957–1972. 10.1098/rstb.2003.1380 PMC1693287 14728791

[mbo3681-bib-0021] Fox, J. , & Weisberg, S . (2011). Car: Companion to applied regression. Available at: http://CRAN.R-project.org/package=car Accessed, 20.

[mbo3681-bib-0022] Gibbons, S. M. , Scholz, M. , Hutchison, A. L. , Dinner, A. R. , Gilbert, J. A , & Coleman, M. L. (2016). Disturbance regimes predictably alter diversity in an ecologically complex bacterial system. mBio, 7(6), e01372‐16. 10.1128/mBio.01372-16 PMC5181773 27999158

[mbo3681-bib-0023] Göbel, P . (2010). Quellen im Münsterland. Beiträge zur Hydogeologie, Wasserwirtschaft, Ökologie und Didaktik. Bönen, Druck Verlag Kettler.

[mbo3681-bib-0024] Goldscheider, N. , Hunkeler, D. , & Rossi, P. (2006). Microbial biocenoses in pristine aquifers and an assessment of investigative methods. Hydrogeology Journal, 14, 926–941. 10.1007/s10040-005-0009-9

[mbo3681-bib-0025] Gower, J. C. (1966). Some distance properties of latent root and vector methods used in multivariate analysis. Biometrika, 53, 325 10.1093/biomet/53.3-4.325

[mbo3681-bib-0026] Graham, E. B. , Knelman, J. E. , Schindlbacher, A. , Siciliano, S. , Breulmann, M. , Yannarell, A. , … Nemergut, D. R . (2016). Microbes as engines of ecosystem function: When does community structure enhance predictions of ecosystem processes? Frontiers in Microbiology, 7, 214. 26941732 10.3389/fmicb.2016.00214 PMC4764795

[mbo3681-bib-0027] Griebler, C. , & Mösslacher, F. (2003). Grundwasser‐Ökologie. Vienna: Facultas UTB.

[mbo3681-bib-0028] Griffiths, B. S. , Ritz, K. , Bardgett, R. D. , Cook, R. , Christensen, S. , Ekelund, F. , … Nicolardot, B. (2000). Ecosystem response of pasture soil communities to fumigation‐induced microbial diversity reductions: An examination of the biodiversity‐ecosystem function relationship. Oikos, 90, 279–294. 10.1034/j.1600-0706.2000.900208.x

[mbo3681-bib-0029] Haag, D. , & Kaupenjohann, M. (2001). Landscape fate of nitrate fluxes and emissions in Central Europe ‐ A critical review of concepts, data, and models for transport and retention. Agriculture Ecosystems & Environment, 86, 1–21. 10.1016/S0167-8809(00)00266-8

[mbo3681-bib-0030] Hafouzov, B . (2010). Hydrochemie des Grund‐ und Quellwassers in den Baumbergen In Göbel & P (Ed.) Abhandlungen aus dem Westfälischen Museum für Naturkunde, 72. Jahrgang, Heft 3/4. Bönen: Druck Verlag Kettler.

[mbo3681-bib-0031] Hanshew, A. S. , Mason, C. J. , Raffa, K. F. , & Currie, C. R. (2013). Minimization of chloroplast contamination in 16S rRNA gene pyrosequencing of insect herbivore bacterial communities. Journal of Microbiological Methods, 95, 149–155. 10.1016/j.mimet.2013.08.007 23968645 PMC4133986

[mbo3681-bib-0032] Hibbing, M. E. , Fuqua, C. , Parsek, M. R. , & Peterson, S. B. (2010). Bacterial competition: Surviving and thriving in the microbial jungle. Nature Reviews Microbiology, 8, 15–25. 10.1038/nrmicro2259 19946288 PMC2879262

[mbo3681-bib-0033] Hofmann, H. , & Cartwright, I. (2013). Using hydrogeochemistry to understand inter‐aquifer mixing in the on‐shore part of the Gippsland Basin, southeast Australia. Applied Geochemistry, 33, 84–103. 10.1016/j.apgeochem.2013.02.004

[mbo3681-bib-0034] Hothorn, T. , Bretz, F. , & Westfall, P. (2008). Simultaneous inference in general parametric models. Biometrical Journal, 50, 346–363. 10.1002/(ISSN)1521-4036 18481363

[mbo3681-bib-0035] Ibekwe, A. M. , Ma, J. C. , & Murinda, S. E. (2016). Bacterial community composition and structure in an Urban River impacted by different pollutant sources. Science of the Total Environment, 566, 1176–1185. 10.1016/j.scitotenv.2016.05.168 27267715

[mbo3681-bib-0036] Kaiser, K. , Wemheuer, B. , Korolkow, V. , Wemheuer, F. , Nacke, H. , Schoning, I. , … Daniel, R. (2016). Driving forces of soil bacterial community structure, diversity, and function in temperate grasslands and forests. Scientific Reports, 6, 33696 10.1038/srep33696 27650273 PMC5030646

[mbo3681-bib-0037] Karczewski, K. , Riss, H. W. , & Meyer, E. I. (2017). Comparison of DNA‐fingerprinting (T‐RFLP) and high‐throughput sequencing (HTS) to assess the diversity and composition of microbial communities in groundwater ecosystems. Limnologica, 67, 45–53. 10.1016/j.limno.2017.10.001

[mbo3681-bib-0038] Kim, M. , Heo, E. , Kang, H. , & Adams, J. (2013). Changes in soil bacterial community structure with increasing disturbance frequency. Microbial Ecology, 66, 171–181. 10.1007/s00248-013-0237-9 23681490

[mbo3681-bib-0039] Kindt, R. , & Coe, R . (2005). Tree diversity analysis: A manual and software for common statistical methods for ecological and biodiversity studies, World Agroforestry Centre.

[mbo3681-bib-0040] Kotsyurbenko, O. R. , Glagolev, M. V. , Nozhevnikova, A. N. , & Conrad, R. (2001). Competition between homoacetogenic bacteria and methanogenic archaea for hydrogen at low temperature. FEMS Microbiology Ecology, 38, 153–159. 10.1111/j.1574-6941.2001.tb00893.x

[mbo3681-bib-0041] Kundzewicz, Z. W. , & Döll, P. (2009). Will groundwater ease freshwater stress under climate change? Hydrological Sciences Journal‐Journal Des Sciences Hydrologiques, 54, 665–675. 10.1623/hysj.54.4.665

[mbo3681-bib-0042] Lawrence, D. , Fiegna, F. , Behrends, V. , Bundy, J. G. , Phillimore, A. B. , Bell, T. , & Barraclough, T. G. (2012). Species interactions alter evolutionary responses to a novel environment. PLOS Biology, 10, e1001330 10.1371/journal.pbio.1001330 22615541 PMC3352820

[mbo3681-bib-0043] Liotta, M. , D'Alessandro, W. , Arienzo, I. , & Longo, M. (2017). Tracing the circulation of groundwater in volcanic systems using the 87 Sr/86 Sr ratio: Application to Mt. Etna. Journal of Volcanology and Geothermal Research, 331, 102–107. 10.1016/j.jvolgeores.2017.01.002

[mbo3681-bib-0044] Lockhart, K. M. , King, A. M. , & Harter, T. (2013). Identifying sources of groundwater nitrate contamination in a large alluvial groundwater basin with highly diversified intensive agricultural production. Journal of Contaminant Hydrology, 151, 140–154. 10.1016/j.jconhyd.2013.05.008 23800783

[mbo3681-bib-0045] Lyons, W. , Tyler, S. , Gaudette, H. , & Long, D. (1995). The use of strontium isotopes in determining groundwater mixing and brine fingering in a playa spring zone, Lake Tyrrell, Australia. Journal of Hydrology, 167, 225–239. 10.1016/0022-1694(94)02601-7

[mbo3681-bib-0046] Ma, J. C. , Ibekwe, A. M. , Yang, C. H. , & Crowley, D. E. (2016). Bacterial diversity and composition in major fresh produce growing soils affected by physiochemical properties and geographic locations. Science of the Total Environment, 563, 199–209. 10.1016/j.scitotenv.2016.04.122 27135583

[mbo3681-bib-0047] McLay, C. D. A. , Dragten, R. , Sparling, G. , & Selvarajah, N. (2001). Predicting groundwater nitrate concentrations in a region of mixed agricultural land use: A comparison of three approaches. Environmental Pollution, 115, 191–204. 10.1016/S0269-7491(01)00111-7 11706792

[mbo3681-bib-0048] McMurdie, P. J. , & Holmes, S. (2013). phyloseq: An R package for reproducible interactive analysis and graphics of microbiome census data. PLoS ONE, 8, e61217 10.1371/journal.pone.0061217 23630581 PMC3632530

[mbo3681-bib-0049] Morris, E. K. , Caruso, T. , Buscot, F. , Fischer, M. , Hancock, C. , Maier, T. S. , … Rillig, M. C. (2014). Choosing and using diversity indices: Insights for ecological applications from the German Biodiversity Exploratories. Ecology and Evolution, 4, 3514–3524. 10.1002/ece3.1155 25478144 PMC4224527

[mbo3681-bib-0050] Morris, B. L. , Lawrence, A. R. , Chilton, P. , Adams, B. , Calow, R. C. , & Klinck, B. A . (2003). Groundwater and its susceptibility to degradation: A global assessment of the problem and options for management, United Nations Environment Programme.

[mbo3681-bib-0051] Moya, C. E. , Raiber, M. , Taulis, M. , & Cox, M. E. (2016). Using environmental isotopes and dissolved methane concentrations to constrain hydrochemical processes and inter‐aquifer mixing in the Galilee and Eromanga Basins, Great Artesian Basin, Australia. Journal of Hydrology, 539, 304–318. 10.1016/j.jhydrol.2016.05.016

[mbo3681-bib-0052] Oksanen, J. , Kindt, R. , Legendre, P. , O'Hara, B. , Stevens, M. H. H. , Oksanen, M. J. , & Suggests, M. (2007). The vegan package. Community Ecology Package, 10, 631–637.

[mbo3681-bib-0053] Paradis, E. , Claude, J. , & Strimmer, K. (2004). APE: Analyses of Phylogenetics and Evolution in R language. Bioinformatics, 20, 289–290. 10.1093/bioinformatics/btg412 14734327

[mbo3681-bib-0054] Pedersen, K. (2013). Metabolic activity of subterranean microbial communities in deep granitic groundwater supplemented with methane and H2. The ISME Journal, 7, 839–849. 10.1038/ismej.2012.144 23235288 PMC3603388

[mbo3681-bib-0055] Petchey, O. L. , & Gaston, K. J. (2002). Extinction and the loss of functional diversity. Proceedings of the Royal Society B‐Biological Sciences, 269, 1721–1727. 10.1098/rspb.2002.2073 PMC1691076 12204134

[mbo3681-bib-0056] Peters, N. E. , & Meybeck, M. (2000). Water quality degradation effects on freshwater availability: Impacts to human activities. Water International, 25, 185–193. 10.1080/02508060008686817

[mbo3681-bib-0057] Philippot, L. , Bru, D. , Saby, N. , Čuhel, J. , Arrouays, D. , Šimek, M. , & Hallin, S. (2009). Spatial patterns of bacterial taxa in nature reflect ecological traits of deep branches of the 16S rRNA bacterial tree. Environmental Microbiology, 11, 3096–3104. 10.1111/j.1462-2920.2009.02014.x 19638171

[mbo3681-bib-0058] Piper, C. L. , Siciliano, S. D. , Winsley, T. , & Lamb, E. G. (2015). Smooth brome invasion increases rare soil bacterial species prevalence, bacterial species richness and evenness. Journal of Ecology, 103, 386–396. 10.1111/1365-2745.12356

[mbo3681-bib-0059] Portal‐Celhay, C. , & Blaser, M. J. (2012). Competition and resilience between founder and introduced bacteria in the *Caenorhabditis elegans* gut. Infection and Immunity, 80, 1288–1299. 10.1128/IAI.05522-11 22184417 PMC3294642

[mbo3681-bib-0060] Quast, C. , Pruesse, E. , Yilmaz, P. , Gerken, J. , Schweer, T. , Yarza, P. , … Glockner, F. O. (2013). The SILVA ribosomal RNA gene database project: Improved data processing and web‐based tools. Nucleic Acids Research, 41, D590–D596. 23193283 10.1093/nar/gks1219 PMC3531112

[mbo3681-bib-0061] R Core Team . (2015). R Core Team 2013. R: A language and environment for statistical computing. R Foundation for Statistical Computing, Vienna, Austria. URL http://www.R-project.org.

[mbo3681-bib-0062] Rivett, M. O. , Buss, S. R. , Morgan, P. , Smith, J. W. N. , & Bemment, C. D. (2008). Nitrate attenuation in groundwater: A review of biogeochemical controlling processes. Water Research, 42, 4215–4232. 10.1016/j.watres.2008.07.020 18721996

[mbo3681-bib-0063] Schirmer, C . (2010). Chemisch‐ökologische Untersuchung der Eutrophierung des Berkelquelltopfes in Billerbeck (Kreis Coesfeld, Nordrhein‐Westfalen) In Göbel P. (Ed.), Abhandlungen aus dem Westfälischen Museum für Naturkunde, 72. Jahrgang, Heft 3/4 (pp. 1–2). Bönen: Druck Verlag Kettler.

[mbo3681-bib-0064] Schloss, P. D. , Gevers, D. , & Westcott, S. L. (2011). Reducing the effects of PCR amplification and sequencing artifacts on 16S rRNA‐based studies. PLoS ONE, 6, e27310 10.1371/journal.pone.0027310 22194782 PMC3237409

[mbo3681-bib-0065] Schloss, P. D. , Westcott, S. L. , Ryabin, T. , Hall, J. R. , Hartmann, M. , Hollister, E. B. , … Weber, C. F. (2009). Introducing mothur: Open‐source, platform‐independent, community‐supported software for describing and comparing microbial communities. Applied and Environmental Microbiology, 75, 7537–7541. 10.1128/AEM.01541-09 19801464 PMC2786419

[mbo3681-bib-0066] Scow, K. M. , & Hicks, K. A. (2005). Natural attenuation and enhanced bioremediation of organic contaminants in groundwater. Current Opinion in Biotechnology, 16, 246–253. 10.1016/j.copbio.2005.03.009 15961025

[mbo3681-bib-0067] Shand, P. , Darbyshire, D. , Love, A. , & Edmunds, W. (2009). Sr isotopes in natural waters: Applications to source characterisation and water–rock interaction in contrasting landscapes. Applied Geochemistry, 24, 574–586. 10.1016/j.apgeochem.2008.12.011

[mbo3681-bib-0068] Shiklomanov, I. A. , & Rodda, J. C. (2004). World water resources at the beginning of the twenty‐first century. NY: Cambridge University Press.

[mbo3681-bib-0069] Staley, C. , & Sadowsky, M. J. (2016). Regional similarities and consistent patterns of local variation in beach sand bacterial communities throughout the northern hemisphere. Applied and Environmental Microbiology, 82, 2751–2762. 10.1128/AEM.00247-16 26921429 PMC4836431

[mbo3681-bib-0070] Tardy, V. , Spor, A. , Mathieu, O. , Leveque, J. , Terrat, S. , Plassart, P. , … Maron, P. A. (2015). Shifts in microbial diversity through land use intensity as drivers of carbon mineralization in soil. Soil Biology & Biochemistry, 90, 204–213. 10.1016/j.soilbio.2015.08.010

[mbo3681-bib-0071] Tilman, D. , Fargione, J. , Wolff, B. , D'Antonio, C. , Dobson, A. , Howarth, R. , … Swackhamer, D. (2001). Forecasting agriculturally driven global environmental change. Science, 292, 281–284. 10.1126/science.1057544 11303102

[mbo3681-bib-0072] Turlapati, S. A. , Minocha, R. , Bhiravarasa, P. S. , Tisa, L. S. , Thomas, W. K. , & Minocha, S. C. (2013). Chronic N‐amended soils exhibit an altered bacterial community structure in Harvard Forest, MA, USA. FEMS Microbiology Ecology, 83, 478–493. 10.1111/1574-6941.12009 22974374

[mbo3681-bib-0073] Vuono, D. C. , Benecke, J. , Henkel, J. , Navidi, W. C. , Cath, T. Y. , Munakata‐Marr, J. , … Drewes, J. E. (2015). Disturbance and temporal partitioning of the activated sludge metacommunity. ISME Journal, 9, 425–435. 10.1038/ismej.2014.139 25126758 PMC4303635

[mbo3681-bib-0074] Wick, K. , Heumesser, C. , & Schmid, E. (2012). Groundwater nitrate contamination: Factors and indicators. Journal of Environmental Management, 111, 178–186. 10.1016/j.jenvman.2012.06.030 22906701 PMC3482663

[mbo3681-bib-0075] Wright, J. , Kirchner, V. , Bernard, W. , Ulrich, N. , McLimans, C. , Campa, M. F. , … Mackelprang, R. (2017). Bacterial community dynamics in dichloromethane‐contaminated groundwater undergoing natural attenuation. Frontiers in Microbiology, 8, 2300 10.3389/fmicb.2017.02300 29213257 PMC5702783

[mbo3681-bib-0076] Zhang, S. H. , Pang, S. , Wang, P. F. , Wang, C. , Guo, C. , Addo, F. G. , & Li, Y. (2016). Responses of bacterial community structure and denitrifying bacteria in biofilm to submerged macrophytes and nitrate. Scientific Reports, 6, 36178 10.1038/srep36178 27782192 PMC5080643

